# Burkitt lymphoma beyond *MYC* translocation: N-MYC and DNA methyltransferases dysregulation

**DOI:** 10.1186/s12885-015-1661-7

**Published:** 2015-10-09

**Authors:** Giulia De Falco, Maria Raffaella Ambrosio, Fabio Fuligni, Anna Onnis, Cristiana Bellan, Bruno Jim Rocca, Mohsen Navari, Maryam Etebari, Lucia Mundo, Sara Gazaneo, Fabio Facchetti, Stefano A. Pileri, Lorenzo Leoncini, Pier Paolo Piccaluga

**Affiliations:** 1 Department of Medical Biotechnologies, University of Siena, Italy - Via delle Scotte, 6 - 53100 Siena, Italy; 2 School of Biological and Chemical Sciences, Queen Mary University of London, London, UK; 3 Department of Experimental, Diagnostic, and Specialty Medicine, University of Bologna, Via Zamboni, 33, 40126 Bologna, Italy; 4 Unit of Pathology, Brescia University, Piazza del Mercato, 15, Brescia, Italy

## Abstract

**Background:**

The oncogenic transcription factor *MYC* is pathologically activated in many human malignancies. A paradigm for *MYC* dysregulation is offered by Burkitt lymphoma, where chromosomal translocations leading to Immunoglobulin gene*-MYC* fusion are the crucial initiating oncogenic events. However, Burkitt lymphoma cases with no detectable *MYC* rearrangement but maintaining MYC expression have been identified and alternative mechanisms can be involved in *MYC* dysregulation in these cases.

**Methods:**

We studied the microRNA profile of *MYC* translocation-positive and *MYC* translocation-negative Burkitt lymphoma cases in order to uncover possible differences at the molecular level. Data was validated at the mRNA and protein level by quantitative Real-Time polymerase chain reaction and immunohistochemistry, respectively.

**Results:**

We identified four microRNAs differentially expressed between the two groups. The impact of these microRNAs on the expression of selected genes was then investigated. Interestingly, in *MYC* translocation-negative cases we found over-expression of DNA-methyl transferase family members, consistent to hypo-expression of the hsa-miR-29 family. This finding suggests an alternative way for the activation of lymphomagenesis in these cases, based on global changes in methylation landscape, aberrant DNA hypermethylation, lack of epigenetic control on transcription of targeted genes, and increase of genomic instability. In addition, we observed an over-expression of another *MYC* family gene member, *MYCN* that may therefore represent a cooperating mechanism of MYC in driving the malignant transformation in those cases lacking an identifiable *MYC* translocation but expressing the gene at the mRNA and protein levels.

**Conclusions:**

Collectively, our results showed that *MYC* translocation-positive and *MYC* translocation-negative Burkitt lymphoma cases are slightly different in terms of microRNA and gene expression. *MYC* translocation-negative Burkitt lymphoma, similarly to other aggressive B-cell non Hodgkin’s lymphomas, may represent a model to understand the intricate molecular pathway responsible for *MYC* dysregulation in cancer.

**Electronic supplementary material:**

The online version of this article (doi:10.1186/s12885-015-1661-7) contains supplementary material, which is available to authorized users.

## Background

Burkitt lymphoma (BL) is a highly aggressive B-cell non-Hodgkin lymphoma characterized by peculiar clinical, morphological, immunophenotypical, cytogenetic, and gene expression profile features [1]. The current World Health Organization (WHO) classification of tumors of hematopoietic and lymphoid tissue assesses that no single parameter can be used as the gold standard to achieve the diagnosis but that a combination of clinical, histological, immunophenotypical and genetic criteria is necessary [1]. The presence of the *MYC*-associated translocation [t(8;14) *MYC*/Immunoglobulin heavy chain gene (*IGH*)] or variants is necessary to confirm all but the most classic cases. However, in the cases of otherwise typical BL, in which an evident *MYC* translocation cannot be detected by the standard procedures, the diagnosis of BL can still be made [1]. Five to ten percent of BL cases show no translocation, both by classical cytogenetics and molecular methods like fluorescence *in situ* hybridization (FISH) analysis [2, 3]. This may be due to technical failure of FISH, as these cases may present with a very small excision of *MYC* and insertion of the gene into one of the *IG loci,* which is missed by the available probes [4]. Another option is that the breakpoint is localized far outside the region covered by the currently available FISH probes [4]. Even though none of the techniques currently used to diagnose genetic changes can unambiguously rule out all of *MYC* translocations [4], some observations suggest that mechanisms other than translocation are responsible for elevated MYC protein expression in BL even in the absence of genomic rearrangements [5, 6]. Amplification, rearrangement or hypomethylation of the *MYC* oncogene are genetic alterations frequently occurring in many cancers, as carcinoma of the cervix, colon, breast, lung and stomach [7–11], and causing *MYC* to be activated and over-expressed. Previous studies, by integrating structural and functional genomics to catalogue the broad of somatic mutations in BL [12–14] have found that the most mutated gene in BL is *MYC* itself (70 % of cases approximately). Moreover, there is increasing evidence that MYC protein over-expression may occur in tumors without apparent gene alterations [15] and it has been suggested that a dysregulated expression of microRNAs (miRNAs) may represent one of the mechanisms leading to *MYC* overexpression in BL cases lacking a classical *MYC* translocation, through either a direct or indirect mechanism [5, 6]. In recent years, lymphoma studies have uncovered various mechanisms by which miRNAs influence their target genes [16] and it has become clear that alterations in the expression of miRNAs contribute to the pathogenesis of most, if not all, human malignancies [17].

All the mechanisms leading to MYC over-expression, affect the expression of its downstream target genes that are involved in various cellular processes such as cell proliferation, cell growth, apoptosis, differentiation, and stem-cell self-renewal, presumably through DNA over-replication [18]. In addition, *MYC* amplifies the existing gene expression program and can also control global chromatin structure by regulating histone acetylation [19].

Increasing information identifies other essential pathways that are activated in the pathogenesis of BL and highlights the fact that *MYC* translocation alone is insufficient to drive lymphomagenesis. Therefore BL cases lacking the typical *MYC* translocation, but expressing MYC at the protein level, may represent a good model for a more detailed description of *MYC* regulation. In this paper we investigated the microRNA profile of *MYC* translocation-positive and *MYC* translocation-negative BL cases in order to uncover possible differences at the molecular level. We found that *MYC* translocation-positive and -negative BL cases are slightly different in terms of microRNA and gene expression, and we validated our findings at the mRNA and protein levels. Interestingly, in *MYC* translocation-negative BLs we found over-expression of DNA methyltransferase (*DNMT)* family members, consistent to hypo-expression of hsa-miR-29 family. This finding suggests an alternative way for the activation of lymphomagenesis in these cases, based on global changes in methylation landscape, aberrant DNA hypermethylation, lack of epigenetic control on transcription of targeted genes, and increase of genomic instability. In addition, we observed the over-expression of another *MYC* family gene member, *MYCN* that may therefore represent an additional mechanism for malignant transformation.

Our findings may be helpful to explain the pathogenetic mechanisms of tumors in which overexpression of *MYC* is independent of a chromosomal translocation or a gene amplification.

## Methods

### Ethics

This study was approved by the ethics committee of the University of Siena, Italy and of Lacor Hospital, Uganda. Study participants or their legal guardians provided written informed consent.

### Case selection

109 Burkitt lymphoma cases, enrolled in the International Network for Cancer Treatment and Research (INCTR) study on African BL, were used for this study. All cases were recorded in childhood and diagnosed as BL by an expert panel on histological slides stained with Haematoxylin and Eosin (H&E) and Giemsa, and by immunophenotyping, according to the WHO classification [1, 20]. Ten cases did not show the typical t(8;14), t(8;2) and t(8;22) *MYC*-translocations at FISH analysis (*MYC* translocation negative in the following) by using both dual-fusion probes and split-signal probes for *IGH* and Immunoglobulin light chain gene (*IGL*) *loci* as well as an LSI *IGH*/*MYC* CEP 8 Tri-color dual-fusion probe (Vysis, Abbott Molecular IL, USA). FISH analysis using *BCL2* and *BCL6* probes was also negative. All cases were otherwise completely typical in term of clinical presentation (age: median 7, range 4–10; female/male ratio: 4/6; nodal/extra-nodal ratio: 2/8), morphology and immunophenotype (CD10+, BCL6+, BCL2-, CD38+, CD44-, Ki-67 100 %) to make a diagnosis of BL.

The analysis of the EBV status was performed by *in situ* hybridization for EBV-encoded RNA (EBER) as previously reported [6]. In particular, 8/10 *MYC* translocation negative cases were EBV-positive, whereas the positivity to the virus was detected in 90 % of *MYC* translocation positive cases.

Unfortunately, RNA extracted from formalin-fixed and paraffin-embedded (FFPE) material precluded next generation sequencing (NGS) studies in most cases, which was therefore performed only in one case, whose fresh tissue was available.

### RNA extraction

For gene expression analysis, RecoverAll™ Total Nucleic Acid Isolation Kit (Life Technologies, Carlsbad, California, USA) was used to extract total RNA from FFPE tissues. Up to five 10 μm sections were processed per reaction. FFPE samples were deparaffinised using a series of xylene and ethanol washes. Next, they were subjected to a rigorous protease digestion with an incubation time tailored for recovery of total RNA. RNA was purified using a rapid glass-fiber filter methodology that includes an on-filter DNAse treatment and were eluted into the low salt buffer provided. On the other hand, for miRNA analysis RNA was extracted from FFPE sections of primary tumors and reactive lymph nodes using the miRNeasy FFPE Kit (Qiagen, Milan, Italy), according to the manufacturer’s instructions.

The amount and quality of RNA were evaluated by measuring the OD at 260 nm and the 260/230 and 260/280 ratios using a Nanodrop spectrophotometer (Celbio, Milan, Italy). The quality of RNA was also checked using a Bioanalyzer 2100 (Agilent, CA, USA).

### Next generation sequencing

High-throughput RNA sequencing produced about 66 million of 75 bp paired ends reads (theoretical coverage calculated on Ref Seq transcriptome 84X). Chromosomal translocations were detected using a bioinformatic pipeline that combines results from three different fusion-detection tools (deFuse, Chimerascan and Tophat Fusion) [21–23] and filtered on non-tumor controls using previously sequenced control reactive lymph nodes. *MYC* gene expression was estimated in one *MYC* translocation*-*negative sample and in other 21 endemic Burkitt lymphomas using the transcripts parts per million (TPM) calculation method [24].

Single Nucleotide Variants (SNVs) and short insertions and deletions (Indels) were called using the Genome Analysis Toolkit (GATK) [25] after mapping quality score recalibration and local realignment around indels. All of the mutations detected were filtered using tresholds based on quality, coverage and strand of the mapped reads and according to variants already present in public databases (Hapmap, dbSNP and 1000genome project) [26]. The Annovar tool [27] was used for functional annotation of variants, including exonic functions and aminoacid changes. All the mutations found in the *MYC* gene, including variations in intergenic, intronic and UTR regions, were manually checked and explorated using the Integrative Genomic Viewer 2.03 (IGV) visualization tool [28].

### MicroRNA array profiling

MiRNA profiling was performed by an external facility (Exiqon, Copenhagen, Denmark). The samples were labelled using the miRCURY™ Hy3/Hy5 Power labelling kit and hybridized on the miRCURY™ LNA Array (5^th^ Generation arrays, *hsa*, *mmu* and *rno*, Exiqon).

Raw data was then received and analyzed in our laboratories. Briefly, signals quantified by microarrays were processed with a normalization pipeline using MIDAS v2.22 software [29]: bad channels (intensity values less than 1) were filtered prior to normalization, and all the spots with a signal/noise value less than 2 were marked as “bad” and excluded from analysis (background correction). Signals were normalized using the global Lowess (Locally weighted scatterplot smoothing) regression algorithm [30] with a smooth parameter of 0,33, which has been found to produce the best within-slide normalization to minimize the intensity-dependent differences between the dyes. Statistical Analysis was performed using MeV v4.7.4 on a dataset including only human miRNA annotated on miRBase [31]. Unsupervised hierarchical clustering on dataset was used on Pearson correlation of log2(Hy3/Hy5) intensities and all of the samples and miRNAs were clusterized using average linkage method. Principal Component Analysis (PCA) was also used to discriminate the different biological samples on the basis of the distances of a reduced set of new variables (Principal Components). Differentially expressed miRNAs between the two groups (*MYC* translocation-positive *versus MYC* translocation-negative) were identified with a two-tailed *T*-test with Welch approximation for different variance among groups and with different stringency criteria for false discovery rate (adjusted Bonferroni correction and no correction). Results of the test were filtered considering as differentially expressed only miRNAs with adjusted *p*-value less than 0,05 and fold change in absolute value greater than 1 [fold change = mean (group A) - mean(group B)].

### Quantitative Real-Time Polymerase Chain Reaction (RT-qPCR)

Quantitative RT-PCR was performed to validate results of both miRNA and gene expression profiling, and to assess relative expression of *MYC* in ten *MYC* translocation positive and ten *MYC* translocation negative cases. For validation of differentially expressed miRNAs identified by profiling, RNA samples were reverse transcribed using the Universal cDNA synthesis kit (Exiqon, Copenhagen, Denmark), according to the manufacturer’s instructions. RT-qPCR amplification was performed using microRNA LNA™ PCR primer sets (Exiqon, Copenhagen, Denmark) specific for hsa-miR-29a-b, hsa-miR-513a-5p, and hsa-miR-628-3p, and using hsa-Let-7c as a reference gene. Validation of genes potentially targeted by the differentially expressed miRNAs (DNA (cytosine-5)-methyltransferase 1 (*DNMT1*), 3 alpha (*DNMT3A*), 3 beta (*DNMT3B*) was also carried out by RT-qPCR using FluoCycle SYBR green (Euroclone, Celbio, Italy) in 10 *MYC*-translocation positive and 10 *MYC*-translocation negative cases according to manufacturer’s instructions. Non-neoplastic lymph nodes were meant as a negative control; *HPRT* was used as housekeeping gene. Primer sequences were designed using Primer-BLAST [32] and are reported in Table 1. Differences in gene expression were calculated using the ΔΔCt method [33].

**Table 1:** Primers used for RTqPCR. Primer sequences for *DNMT1* amplified a region of 88 bp. Primers for *DNMT3a* amplified a region of 68 bp; Primers for *DNMT3b* amplified a region of 68 bp; Primers for *MYC* amplified a region of 129 bp; Primers for HPRT amplified a region of 191 bp

Gene	Primer sequence
*DNMT1-*FORWARD	5’-CGACTACATCAAAGGCAGCAACCTG-3’
*DNMT1-*REVERSE	5’-TGGAGTGGACTTGTGGGTGTTCTC-3’
*DNMT3A*-FORWARD	5’-TAT TGATGAGCGCACAAGAGAGC-3’
*DNMT3A*-REVERSE	5’-GGGTGTTCCAGGGTAACATTGAG-3’
*DNMT3b*-FORWARD	5’-GGCAAGTTCTCCGAGGTCTCTG-3’
*DNMT3b*-REVERSE	5’-TGGTACATGGCTTTTCGATAGGA-3’
*MYC*-FORWARD	5’-AGCGACTCTGAGGAGGAAC-3’
*MYC*-REVERSE	5’-TGTGAGGAGGTTTGCTGTG-3’
*HPRT-*FORWARD	5’-AGCCAGACTTTGTTGGATTTG-3’
*HPRT*-REVERSE	5’-TTTACTGGCGATGTCAATAAG-3’

### Immunohistochemistry

Immunohistochemistry analysis for MYC (Abcam; dilution 1:200), DNMT1 (BD Biosciences: dilution 1:50), DNMT3A (Abcam; dilution 1:100), DNMT3B (Imgenex; dilution 1:200) and NMYC (ThermoScientific; dilution:1:100) was performed on Bond III automated immunostainer (Leica Microsystem, Bannockburn, IL, USA), with controls in parallel. No epitope retrieval was exploited. Ultravision Detection System using anti-Polyvalent HRP (LabVision, Fremont, CA, USA) and diaminobenzidine (DAB, Dako, Milan-Italy) as a chromogen was employed. The expression level of the proteins was evaluated in the ten *MYC* translocation-positive and ten *MYC* translocation-negative cases used for the RT-qPCR analysis, to validate results. Immunoreactivity was assessed by two investigators and cases with discrepancy were re-viewed to obtain a concordance ratio of more than 90 %. It is noteworthy that the definition of MYC positivity by immunohistochemistry is not universally standardized. However, the literature reports that having at least 40 % of malignant lymphocytes with nuclear MYC expression is considered positive [34]; therefore we used this cut-off to discriminate positive and negative cases. For DNMT1 and DNMT3A, the cut-off level was based on modified Choi et al. system considering only the proportion of neoplastic cells showing a nuclear positivity [35]. The expression of DNMT1, DNMT3A and DNMT3B was considered absent/low if only 0–10 % of tumor cells were stained; intermediate whether the positivity was present in 11–50 % of neoplastic cells, and high when the immmunoreactive cells were >50 %. For N-MYC, only nuclear staining was considered positive with no cut-off level.

## Results

### *MYC* translocation-positive and *MYC* translocation-negative BL cases express MYC at both the mRNA and protein levels

**Fig. 1:**
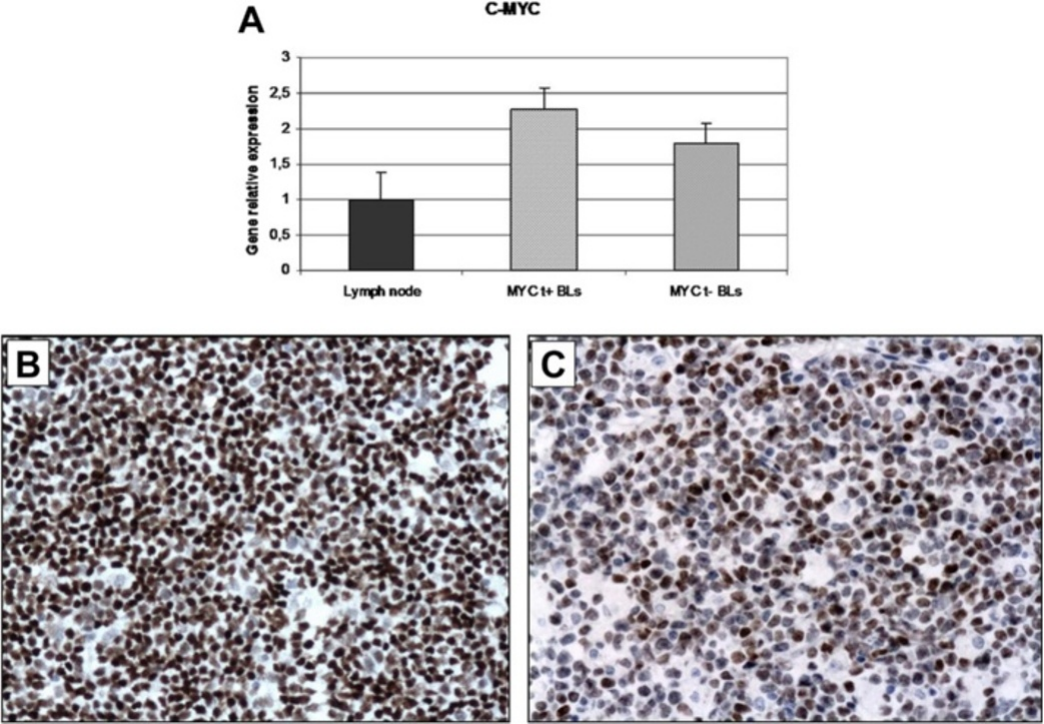
*MYC* mRNA and protein expression in *MYC* translocation-positive and -negative BL cases. **a** Quantitative-RT-PCR. The expression of *MYC* was analysed at the mRNA level in cases either carrying or lacking the translocation. RT-qPCR results show the up-regulation of the gene also in the absence of *MYC* translocation; (**b**-**c**) Immunohistochemistry. In the exemplifying *MYC* translocation-positive case (**b**), a strong staining in about 95 % of neoplastic cells is shown in contrast to the *MYC* translocation-negative one (**c**), in which the staining intensity was present in about 60 % of cells. **b**-**c**: MYC stain. Original Magnification (O.M): 20x

We found that all of *MYC* translocation-positive cases expressed *MYC* at the mRNA and protein levels (Fig. 1a-c). By immunohistochemistry, a strong positivity was observed in about 95 % of neoplastic cells. As far as *MYC* translocation-negative BLs is concerned, we observed that all the 10 samples expressed *MYC* mRNA at variable level (Fig. 1a). The same was true for MYC protein whose positivity was detectable in a percentage of neoplastic cells ranging from 50 to 80 % (Fig. 1b-c). These findings confirmed that *MYC* translocation-negative cases used in this study, even lacking the typical *MYC* translocation, do express the MYC protein (Fig. 1c), suggesting the existence of alternative mechanisms regulating *MYC* expression.

### Next generation sequencing

**Fig. 2:**
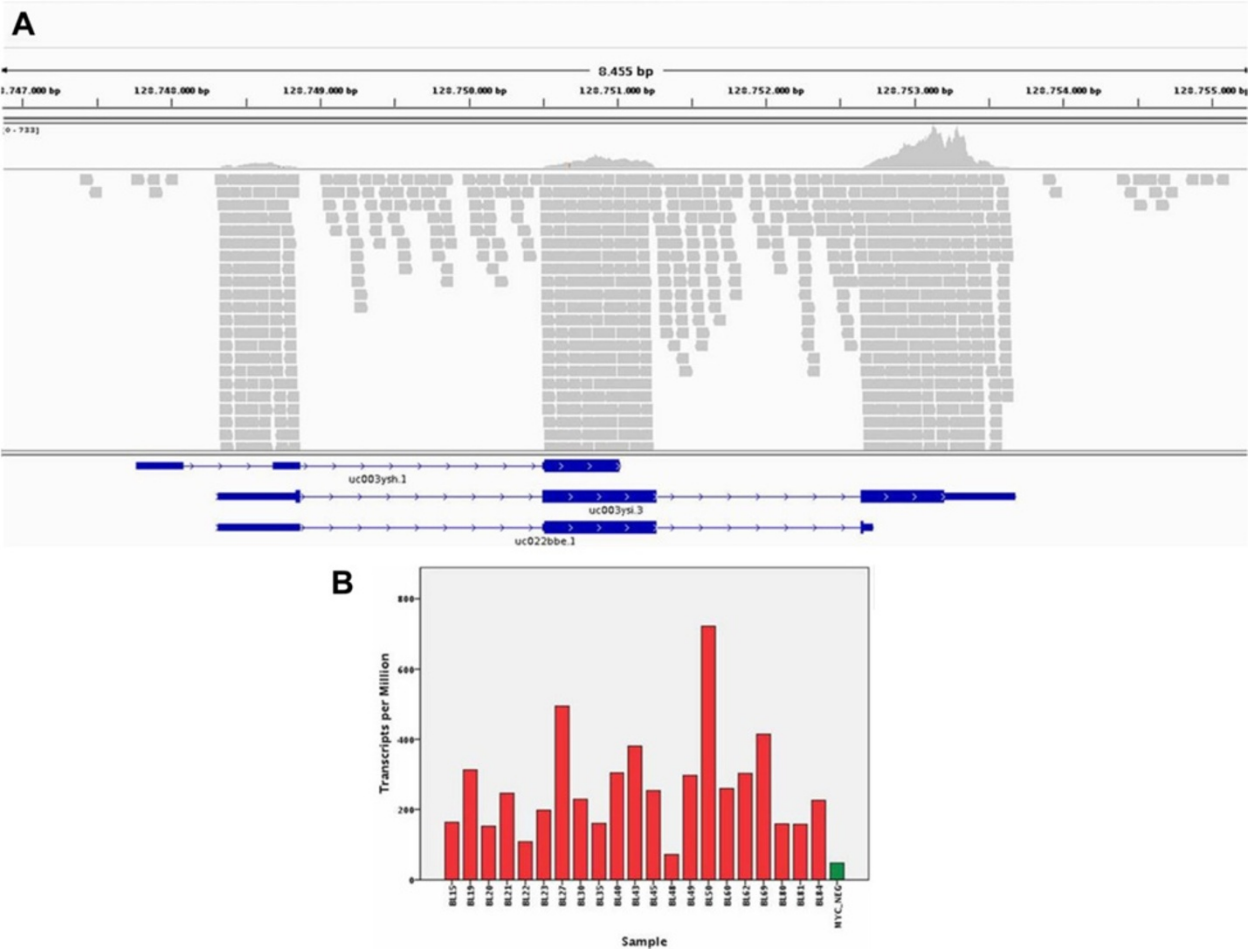
**a** Genomic view of the distribution of *MYC* variants in sequenced sample. Sequence alignments of paired end reads are displayed as greybars spanning exonic sequence of different *MYC* isoforms (blue segments below the reads alignment). Above the reads alignment section, the coverage histogram shows the read depth distribution of the *MYC* gene base per base. **b** Histogram shows the distribution of abundance of the *MYC* gene calculated in transcripts parts per million (TPM), in *MYC* translocation-negative sample (green) and other endemic *MYC* translocation-positive Burkitt lymphomas RNA-seq samples (red)

As we documented *MYC* expression in cases lacking the typical translocation, we sought to verify whether cryptic *MYC* abnormalities might have been missed by FISH analysis. To this aim, we studied by RNA-sequencing the only *MYC* translocation-negative BL case for which adequate material was available. Indeed, analysis of the *MYC locus* revealed a normal structure of *MYC* transcripts (Fig. 2 and Additional file 1: Table S1).

### *MYC* translocation-positive and *MYC* translocation-negative BL cases present with different microRNA expression patterns

**Fig. 3:**
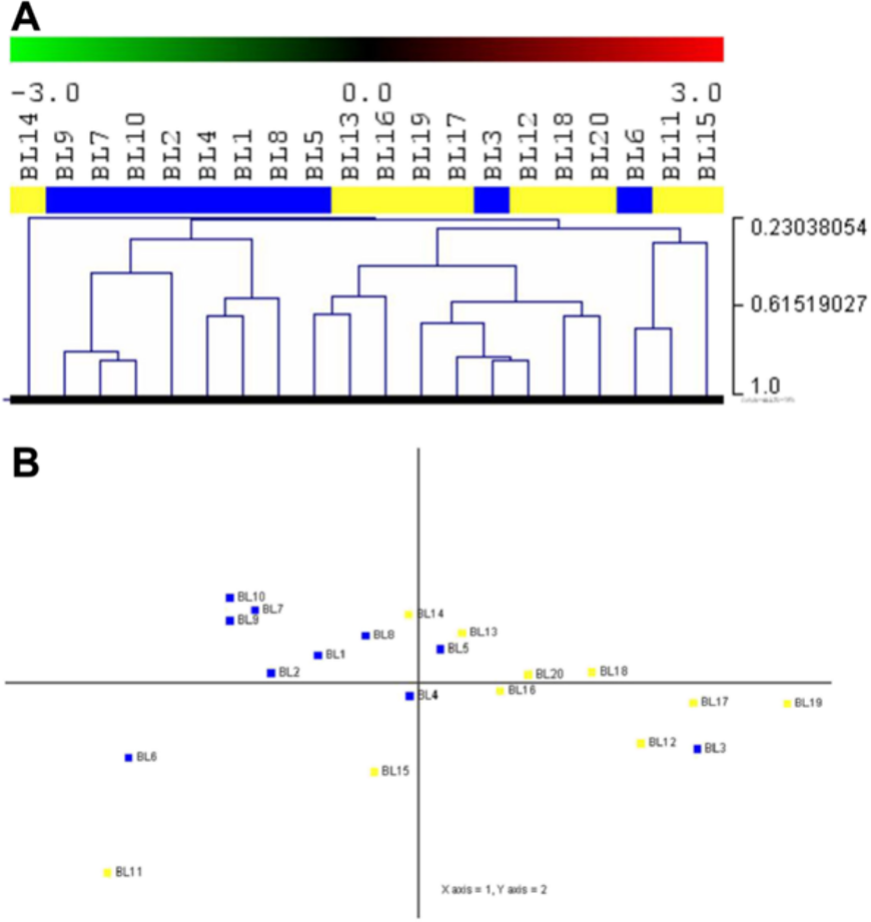
Unsupervised analysis of Burkitt lymphomas. **a** The heat map diagram shows the result of the two-way unsupervised HC of miRNAs and samples based on the expression of 1,375 miRNAs. HC, roughly discriminated *MYC* translocation-negative (yellow) and *MYC* translocation-positive (blue) cases based on the miRNA expression pattern. In the matrix, each row represents a miRNA and each column represents a sample. The color scale illustrates the relative expression level of a miRNA across all samples: red represents an expression level above the mean and green represents expression lower than the mean. **b** PCA confirmed the distinction between MYC translocation-positive (blue) and MYC translocation-negative (yellow) samples

**Fig. 4:**
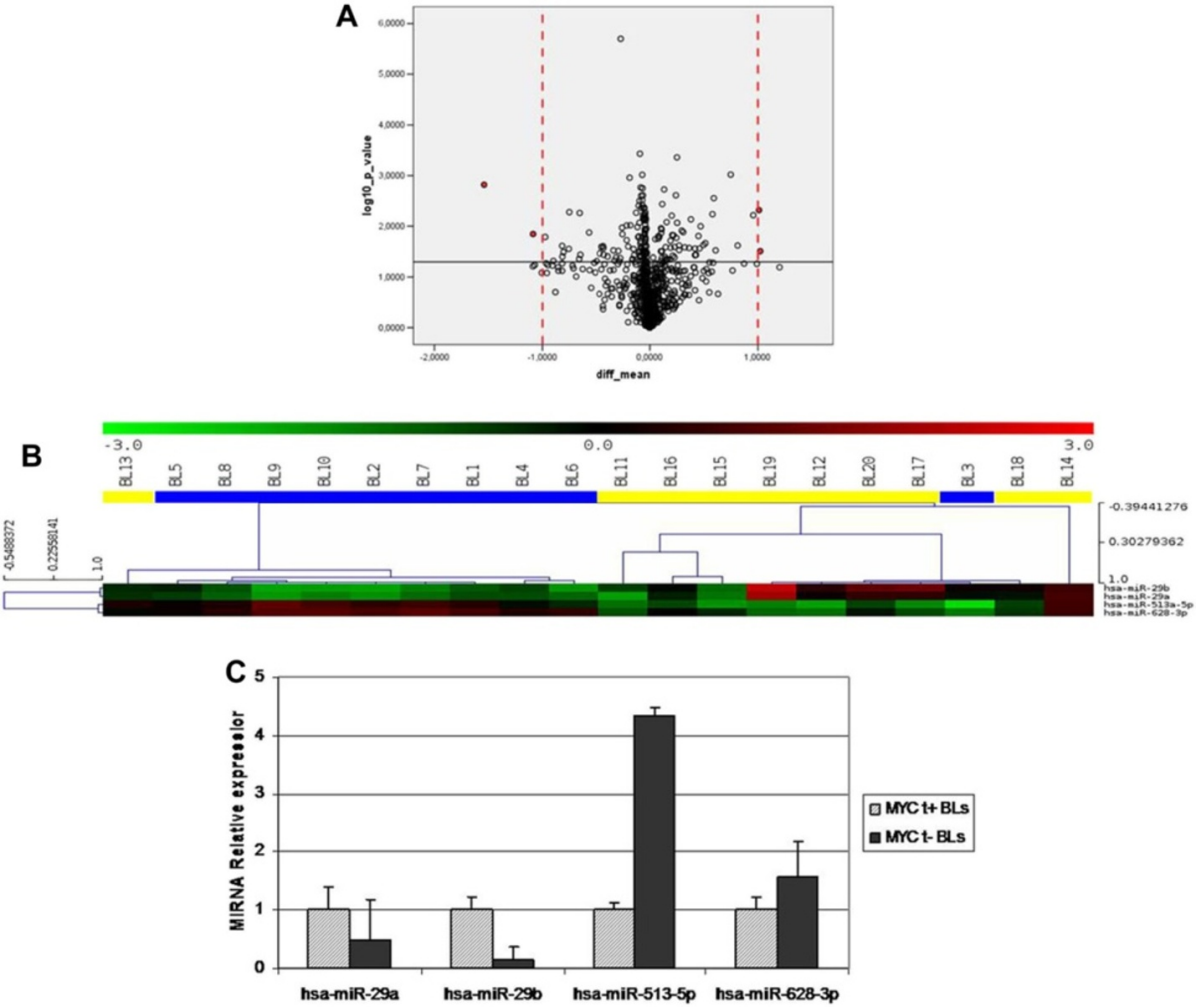
Differentially expressed miRNAs beetween *MYC* translocation-positive and negative Burkitt lymphomas **a** Volcano plot on *T*-test for different miRNA expression between *MYC* translocation-positive and *MYC* translocation-negative. Volcano plot representing filtering threshold for one-tailed *T*-test for differential expression analysis between *MYC* translocation-positive and *MYC* translocation-negative. The plot shows the difference between the means of *MYC* translocation-positive and *MYC* translocation-negative for each miRNA plotted against the negative log10 p-value associated with the *T*-test. Black horizontal shows the threshold for *p*-value = 0,05 and red vertical lines are used for filtering miRNAs on fold change value of 1 and −1. All of the 4 points of the plot highlighted in red represent differentially expressed miRNAs that pass the filtering thresholds on p-value and fold change. **b** Hierarchical clustering on 4 differentially expressed miRNAs between *MYC* translocation-positive and *MYC* translocation-negative. Hierarchical cluster in samples and miRNAs for 4 differentially expressed miRNAs that passed filtering thresholds. Each row represents a miRNA and each column represents a sample. Similar samples and miRNAs of the experiment are connected by a series of branches. The length of each branch represents the distance in terms of Pearson correlation of log2(Hy3/Hy5) between connected samples or miRNAs. The miRNA clustering tree is shown on the left. The color scale shown at the top illustrates the relative expression level of a miRNA across all samples: red represents an expression level above the mean, green represents expression lower than the mean. The samples are colour coded according to the groups; yellow are the *MYC* translocation-positive (BL1-10), blue are the *MYC* translocation-negative. **c** Validation of miRNA profiling was assessed by RT-qPCR, which confirmed differential expression of these miRNAs in the two groups, being hsa-miR-29a and hsa-miR-29b down-regulated and hsa-miR-513a-5p, and hsa-miR-628-3p up-regulated in *MYC* translocation-negative BL cases

To ascertain whether there was a distinctive miRNA signature for *MYC* translocation-positive and negative BLs, we profiled ten *MYC* translocation-positive BLs and ten *MYC* translocation-negative BLs. Unsupervised hierarchical clustering (HC) showed that *MYC* translocation-positive and -negative BLs could be roughly separated in two groups (Fisher exact test, *p* = 0.01) (Fig. 3a). In addition, PCA confirmed the distinction between *MYC* translocation-positive and -negative samples (Fig. 3b). When a supervised approach was adopted, we identified 4 differentially expressed miRNAs out of 894 between *MYC* translocation-positive and -negative BLs, (*T*-test, *p*-values lower than 0.05 and fold change in absolute value greater than 1) (Fig. 4a-b and Table 2). Again, consistently with previous unsupervised analyses, the HC showed a clear distinction between *MYC* translocation-positive and -negative BLs (Fisher exact test, *p* = 0.001).

**Table 2:** miRNA profiling (p-value and fold change)

TargetID	*p* value	Fold change (Absolute value)	Regulation in MYC-neg
hsa-miR-513a-5p	0,031124841	1,02109958	Down
hsa-miR-628-3p	0,004815838	1,01011474	Down
hsa-miR-29a	0,0142882	1,086645638	Up
hsa-miR-29b	0,001516702	1,5403288	Up

By contrast, when we applied the previously described miRNA signature able to discriminate BL from diffuse large B-cell lymphomas (DLBCL) constituted by 30 miRNAs containing *MYC*-regulated and nuclear factor-kB pathways-associated miRNAs [36], we failed to discriminate BL cases according to the presence of *MYC* translocation, this ruling out *bona fide* the possible presence of DLBCLs morphologically mimicking classical BL in the present series (i.e. BL/DLBCL) [1]. Actually, hsa-miR-29b that is up-regulated in DLBCL, is down-regulated also in BL and mostly in *MYC* translocation negative cases.

Validation of the results was performed on all the dysregulated miRNAs so identified (hsa-miR-29a, hsa-miR-29b, hsa-miR-513a-5p, and hsa-miR-628-3p). Quantification of these miRNAs was performed using RT-qPCR in all of the *MYC* translocation-positive and 10 *MYC* translocation-negative cases. Collectively, fold changes of hsa-miR-29a, hsa-miR-29b, hsa-miR-513a-5p, and hsa-miR-628-3p obtained by microarray results were confirmed by RT-qPCR (Fig. 4c). A significant down-regulation of the miR-29 family members was observed in *MYC*-translocation negative cases, whereas the remaining two miRNAs were hyper-expressed in the absence of translocation (*p* < 0.05).

### The microRNA pattern impacts on the gene expression profiling (GEP) of BL cases

After identification of miRNAs discriminating *MYC* translocation-positive and *MYC* translocation-negative samples, we investigated whether they could affect the gene expression pattern of the tumors. 64 putative target genes of such miRNAs were identified by bioinformatics (Additional file 2: Table S2). Interestingly, the 64 predicted miRNA targets turned out to be significantly enriched in molecules involved in gene expression regulation, proliferation, and DNA modification (Additional file 3: Table S3) and included, among others *MYCN* and *DNMT* family members (*1*, *3A*, and *3B*), all known to be involved in malignant transformation.

**Fig. 5:**
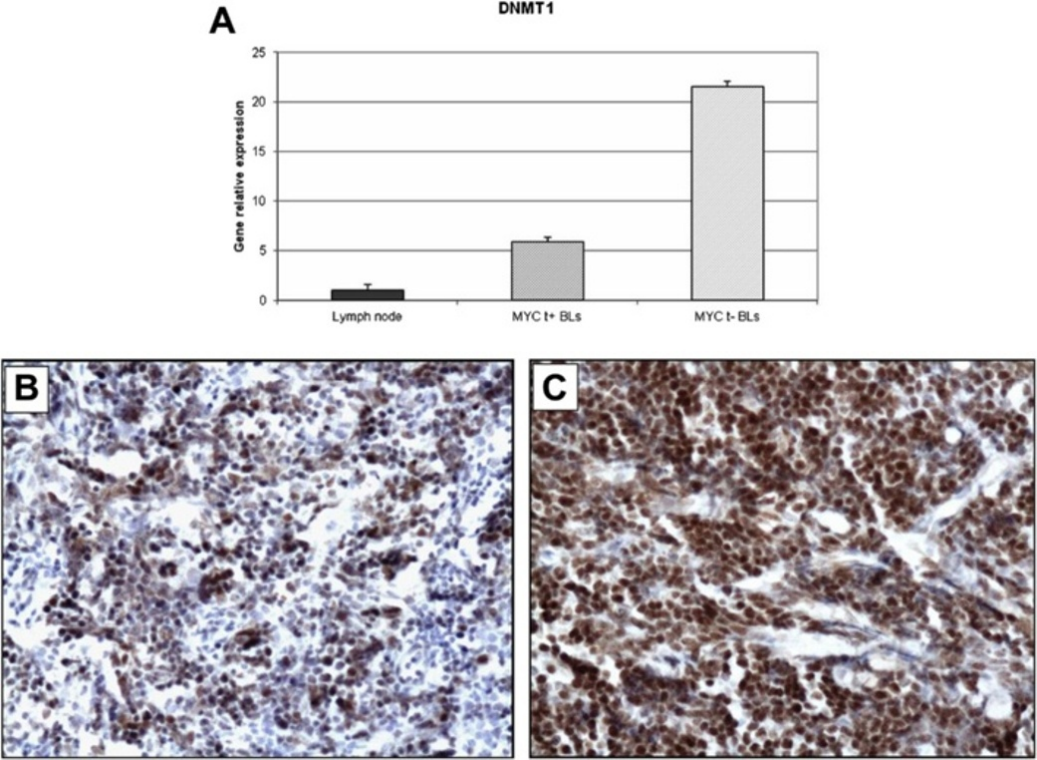
RT-qPCR validation and immunohistochemical evaluation of DNMT1 in *MYC* translocation-positive and *MYC* translocation-negative BL primary tumors. **a** Quantitative-RT-PCR. The expression of DNMT1 was analysed at the mRNA level by RT-qPCR. The results show up-regulation of DNMT1 in cases lacking the translocation; (**b**-**c**) Immunohistochemistry. In the exemplifying *MYC* translocation-positive case (**b**), the staining is present in about 30 % of neoplastic cells, in contrast to the *MYC* translocation-negative one (c), in which the positivity is depicted in about 80 % of cells. **b**-**c**: DNMT1 stain. O.M: 20x

**Fig. 6:**
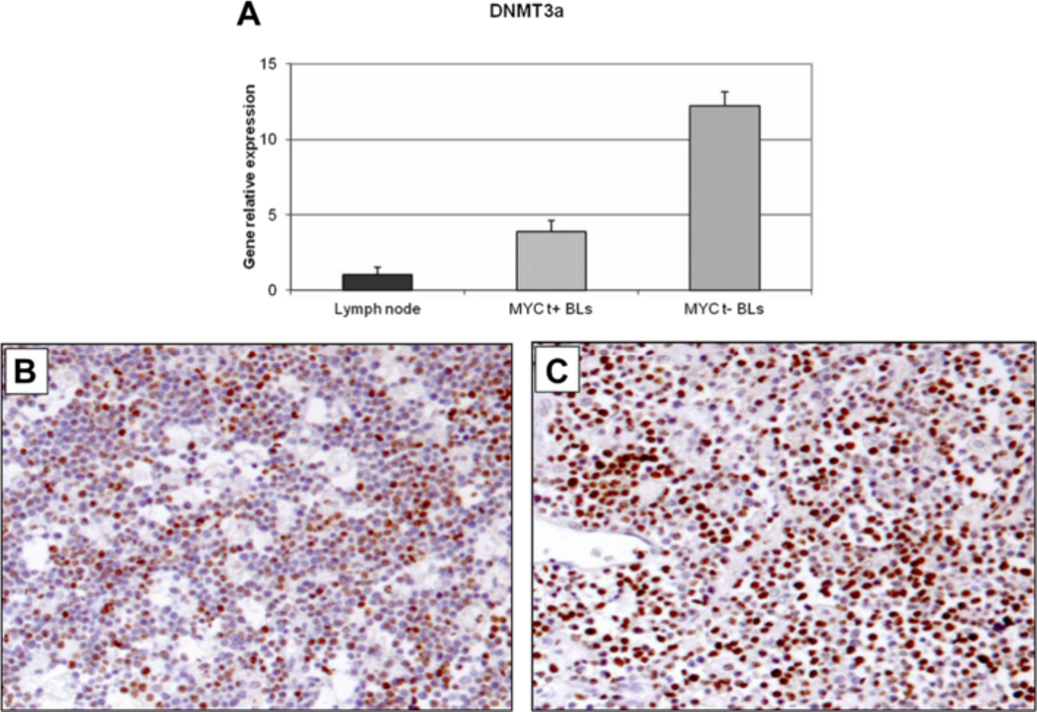
RT-qPCR validation and immunohistochemical evaluation of DNMT3A in *MYC* translocation-positive and *MYC* translocation-negative BL primary tumors. **a** Quantitative-RT-PCR The expression of DNMT3A was analysed at the mRNA level by RT-qPCR. As for DNMT1, DNMT3A resulted up-regulated in cases lacking the translocation; (**b**-**c**) Immunohistochemistry. In the exemplifying *MYC* translocation-positive case (**b**), the staining is shown in 40 % of neoplastic cells in contrast to the *MYC* translocation-negative one (**c**), in which about 60 % of cells are positive. **b**-**c**: DNMT3A stain. O.M: 20x

**Fig. 7:**
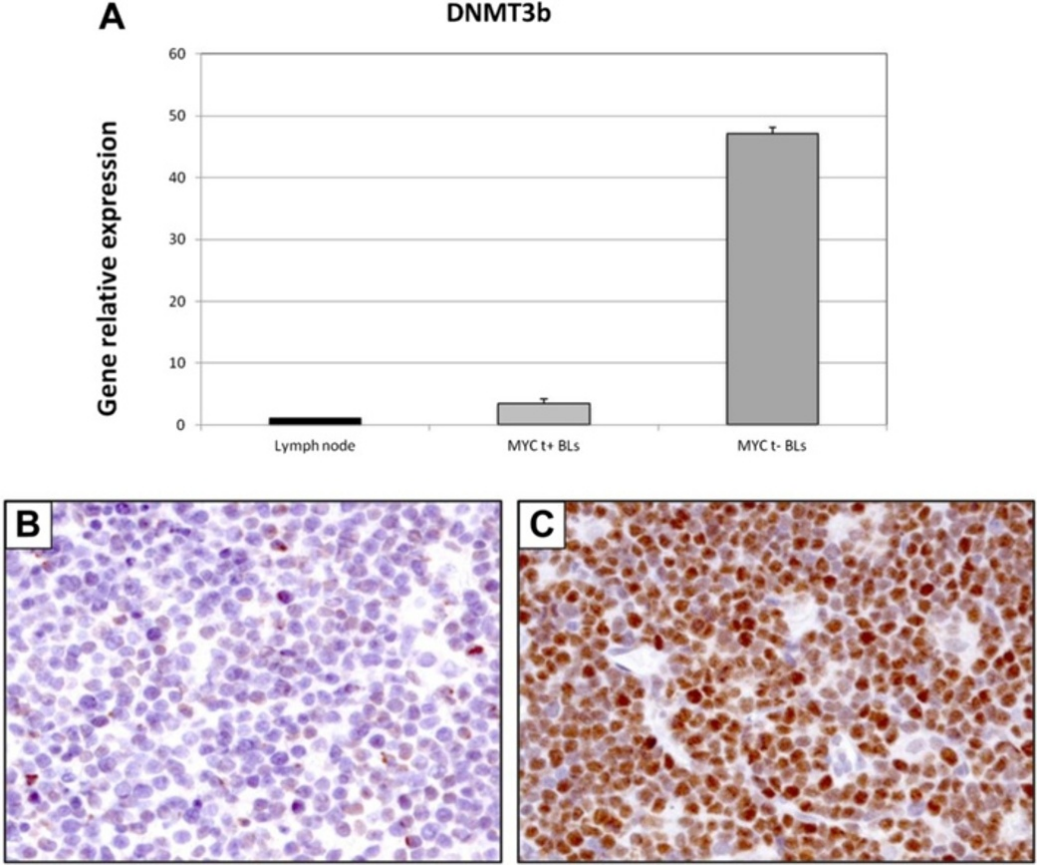
RT-qPCR validation and immunohistochemical evaluation of DNMT3B in *MYC* translocation-positive and *MYC* translocation-negative BL primary tumors. **a** Quantitative-RT-PCR The expression of DNMT3B was analysed at the mRNA level by RT-qPCR. As for DNMT3A, DNMT3B resulted up-regulated in cases lacking the translocation; (**b-c**) Immunohistochemistry. In the exemplifying *MYC* translocation-positive case (**b**), the staining is shown in 5 % of neoplastic cells in contrast to the *MYC* translocation-negative one (**c**), in which about 70 % of cells are positive. **b**-**c**: DNMT3B stain. O.M: 20x

**Fig. 8:**
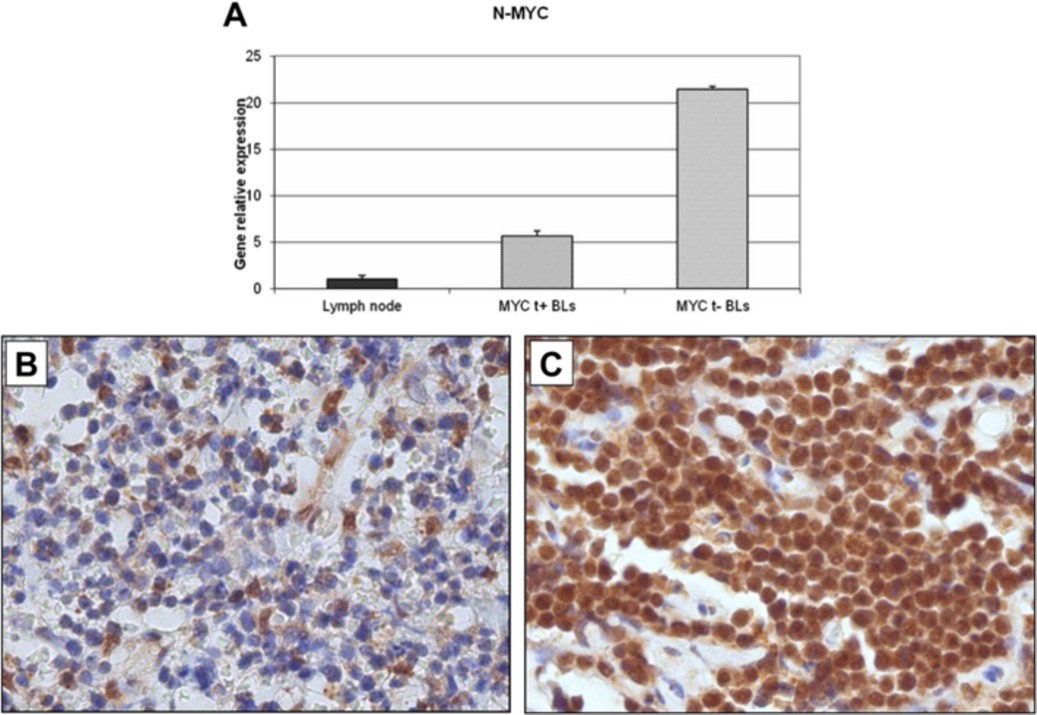
RT-qPCR validation and immunohistochemical evaluation of N-MYC in *MYC* translocation-positive and *MYC* translocation-negative BL primary tumors. **a** Quantitative-RT-PCR The expression of NMYC was analysed RT-qPCR. *MYC*-translocation negative cases show a dramatic hyper-expression of the gene; altogether RT-qPCR results confirmed the bioinformatics predictions, which suggest a regulation of these by the miR29 family. Over-expression of the selected genes is in accordance with down-regulation of the miR-29 family observed in *MYC*-translocation negative cases; (**b-c**) Immunohistochemistry. In the exemplifying *MYC* translocation-positive case (**b**), the staining is present only in 5 % of neoplastic cells in contrast to the *MYC* translocation-negative one (**c**), in which the positivity is detectable in about 90 % of cells. **b**: H&E, **c**: NMYC stain. **b**-**c**, O.M: 40x

Since a direct regulation of *DNMT* family members and *MYCN* by hsa-miR-29b has been previously demonstrated [36, 37], *DNMT1*, *DNMT3A*, *DNMT3B* and *MYCN* mRNA expression analysis was performed in a total of 10 *MYC* translocation-positive and 10 *MYC* translocation-negative cases by RT-qPCR. Interestingly, increased expression of *DNMT1*, *DNMT3* family members and *MYCN* was observed in *MYC* translocation-negative samples in comparison to the *MYC* translocation-positive cases and the control (Figs. 5a, 6a, 7a, 8a).

### DNMT1, DNMT3A, DNMT3B and NMYC protein expression in BL tumour samples

DNMTs and N-MYC protein expression was analyzed in 10 *MYC* translocation-positive and 10 *MYC* translocation-negative BL tumor samples by immunohistochemistry. In *MYC* translocation-positive BLs the positivity for DNMT1 was low/intermediate and ranged from 10 % to 30 % (Fig. 5b). In *MYC* translocation-negative cases, the expression of the protein was high; all the cases showed more than 70 % positive cells (Fig. 5c). DNMT3A protein staining was high in both *MYC* translocation-positive and -negative BLs. However, only 3 out of 10 samples had a percentage of positive cells more than 40 % (Fig. 6b) whereas all *MYC* translocation-negative BLs had more than 60 % of neoplastic cells depicted by the antibody (Fig. 6c). In *MYC* translocation-positive BLs the positivity for DNMT3B was very low and ranged from 5 % to 10 % (Fig. 7b). In *MYC* translocation-negative cases, the expression of the protein was high; all the cases showed more than 70 % positive cells (Fig. 7c). N-MYC protein expression was low in all the *MYC* translocation-positive BLs examined in which the staining was positive in about 5 % of neoplastic cells (Fig. 8b). *MYC*-translocation negative samples demonstrated higher N-MYC positivity that was present in more than 90 % of neoplastic cells (Fig. 8c).

## Discussion

BL is an aggressive B-cell lymphoma with a characteristic clinical presentation, morphology and immunophenotype [1]. Over the past years, the typical translocation, involving the *MYC* oncogene and its variants, has been considered the molecular hallmark of this tumor. However, transcriptional and genomic profiling aimed to distinguish BL *versus* DLBCL revealed the existence of BLs without evident *MYC* translocation clustering with molecular BL. A recent paper reported that BLs lacking *MYC* translocation share a peculiar pattern of chromosome 11q aberration [38]. The significantly lower expression of *MYC* in such cases supported the view that *MYC* is not genomically activated, and the clinical, morphologic, and molecular characterizations of these cases suggest that they represent a distinct subset of *MYC*-negative high-grade B-cell lymphomas with features resembling but not identical to BL. Yet, these findings do not explain the mechanisms through which some classic BL cases lack the typical genetic translocation involving *MYC* but do express *MYC* at the mRNA and the protein level [5, 6]. Dysregulation of *MYC* expression may be due to additional mechanisms, other than common genomic abnormalities, such as a miRNA imbalance [39, 40]. So far, no data is available concerning the miRNA profile of *MYC* translocation-negative cases, besides the evidences previously reported by our group [5, 6]. In this study, we further explored the miRNA profile of BLs carrying or not the classical translocations involving the *MYC* gene.

Interestingly, when we compared the miRNA profiling of *MYC* translocation-positive *versus MYC* translocation-negative BL cases, we identified four miRNAs differentially expressed, of which hsa-miR-513a-5p and hsa-miR-628-3p were up-regulated and two miR-29 family members (hsa-miR-29a and hsa-miR-29b) were down-regulated in BL cases lacking the *MYC* translocation.

Of note, microarray-based miRNA analysis turned out to be quite specific and robust in this study. In fact, all of the genes tested were successfully validated by RT-qPCR.

Hsa-miR-628-3p and hsa-miR-513a-5p are less referred in the literature, whereas, more is known about the miR-29 family [41]. Interestingly, miR-29 family members have been related to malignant transformation, and it has been demonstrated that their down-regulation contributes to MYC-induced lymphomagenesis *in vivo* and *in vitro* models [42, 43]. Thus, hsa-miR-29 family members down-regulation may represent an appealing possible mechanisms able to determine MYC up-regulation and sustain its expression at mRNA and protein level also in the absence of a translocation. Interestingly, a link between the miR-29 family by *MYC* has been recently reported [44], as repression of miR-29 by *MYC* through a corepressor complex with HDAC3 and EZH2 is observed in aggressive B-cell lymphomas [43]. This miRNA family may represent a novel target for tailored therapies as *in vitro* and mouse studies suggest increasing miR-29 expression by combined inhibition of HDAC3 and EZH2. Such an approach could help treat *MYC*-overexpressing cancers [44]. In addition, it has been recently demonstrated that hsa-miR-29b directly binds to *DNMT3A* and *DNMT3B*, and regulates indirectly *DNMT1* by targeting Sp1, a transactivator of the gene [36, 45]. In this scenario, over-expression of *DNMT* family members, due to hypo-expression of hsa-miR-29 family members, may elicit a role in inducing carcinogenesis [46]. The finding that DNMTs were up-regulated in *MYC* translocation-negative BLs suggests an alternative way for the activation of lymphomagenesis in these cases, based on global changes in methylation landscape and loss of epigenetic control. Hsa-miR29a may favor this process by a synergistic hypermethylating effect [47]. In this regard, future studies exploring the global methylation patterns of BL with or without *MYC* translocation are definitely warranted.

We were also intrigued by the observation that another member of the *MYC* family, *MYCN*, was potentially dysregulated in BL cases lacking *MYC* translocation. Literature reports that MYC and N-MYC possess similar ability to induce cell proliferation and transformation although MYC may be more effective in some contexts*.* Over-expression of specific *MYC* family genes is frequently associated with particular types of human tumors [4]; *MYCN* deregulation is almost exclusively associated with solid tumors and only rarely observed in lymphomas. Nonetheless, both N-MYC and MYC are expressed in pro-B cells, and it has been demonstrated that N-MYC can support normal B-cell development in the absence of MYC [48–50]. Over-expression of either MYC or N-MYC under the control of the B cell-specific Eμ enhancer results in development of pro-B cell lymphomas [51]. Finally, complex *MYCN*/*IGH* translocations frequently arise in mice deficient for p53, showing that, in this genetic background, the endogenous N-MYC can compete with MYC as a pro-B cell oncogenic translocation/amplification target [52]. Based on our findings (i.e. over-expression of N-MYC at the mRNA and protein levels in *MYC* translocation-negative cases) one should hypothesize that in BL cases lacking *MYC* translocation N-MYC may represent an alternative cooperating mechanisms in contributing to malignant transformation. Interestingly, two of the differentially expressed miRNAs (miR-513a-5p and miR-628-3p) have been recently reported dysregulated in human neuroblastomas, in which aberrant expression of *MYCN* is quite common [53, 54]. Of note, miR-628-3p expression seems even to correlate with tumors prognosis in such cases [55]. Altogether this observation suggests that *MYCN* aberrant expression itself may impact gene and microRNA expression pattern in BL cases lacking the typical *MYC* translocation. A large body of evidence has documented the existence of an active cross-talk between *MYC* itself and miRNAs machinery, suggesting the existence of a feedback loop between *MYC* and specific miRNAs [56]. This, in turn, might be the cause of a differential gene expression and of functional alterations of neoplastic cells [40]. The difference in has-miR29 family members expression we detected between MYC translocation-positive and MYC-translocation negative BL samples might be related to the lower MYC protein level among cases lacking the MYC-translocation.

## Conclusions

Our results extend the current knowledge on aggressive B-cell lymphomas presenting with MYC expression but lacking a conventional translocation. The evidences of N-MYC and DNMT family member dysregulation point at a more complex scenario involving *MYC* and other players in BL tumorigenesis, and underline the role of a miRNAs-MYC feedback loop. Therefore, *MYC* translocation-negative BL cases can represent a model to understand the intricate molecular pathways responsible for both *MYC* over-expression and its interaction with complex cellular processes.

## Availability of supporting data

All the data used in this study have been deposited in the Gene Expression Omnibus (GEO) database. The accession number is GSE71471, and the link to freely access to all the information is http://www.ncbi.nlm.nih.gov/geo/query/acc.cgi?acc=GSE71471.

## Additional files

Additional file 1: Table S1. List of inter and intra chromosomal gene fusion detected by fusion-detection pipeline. (XLS 89 kb)
Additional file 2: Table S2. Predicted target genes of miRNAs differentially expressed in *MYC* translocation-positive and -negative BLs. (DOC 50 kb)
Additional file 3: Table S3. Gene set enrichment analysis for gene ontology categories of 64 genes predicted as targets of microRNAs differentially expressed in *MYC* translocation-positive and -negative BLs. (DOC 67 kb)

## Abbreviations

BL: Burkitt lymphoma
DLBCL: Diffuse large B-cell lymphoma
DNMT: DNA methyltransferase
FFPE: Formalin fixed and paraffin embedded
FISH: Fluorescence *in situ* hybridization
GATK: Genome Analysis Toolkit
GEP: Gene expression profiling
HC: Unsupervised hierarchical clustering
IGH: Immunoglobulin heavy chain gene
IGL: Immunoglobulin light chain gene
miRNAs: microRNAs
NGS: Next generation sequencing
PCA: Principal component analysis
RT-qPCR: Quantitative Real-Time polymerase chain reaction
SNVs: Single Nucleotide Variants
WHO: World Health Organization
