# Supplementary material for: Burkitt lymphoma beyond MYC translocation: N-MYC and DNA methyltransferases dysregulation
Source: BMC Cancer. 2015 Oct 9;15:668. doi: 10.1186/s12885-015-1661-7 (PMC4600215; doi:10.1186/s12885-015-1661-7)
Supplement: Additional file 2: Table S2. — Predicted target genes of miRNAs differentially expressed in MYC translocation-positive and -negative BLs. (DOC 50 kb) [file 12885_2015_1661_MOESM2_ESM.doc]

**Supplementary Table 2. Predicted target genes of miRNAs differentially expressed in *MYC* translocation-positive and -negative BLs**

| **TargetID** | **miRNA** | **TargetID** | **miRNA** |
| --- | --- | --- | --- |
| *ADAM12* | miR29b-3p | *GLUL* | miR29a-3p |
| *ADAMTS9* | miR29a-3p | *GRN* | miR29b-3p |
| *ARHGEF12* | hsa-miR-628-3p | *HDAC4* | miR29b-3p |
| *BACE1* | miR29a-3p  miR29b-3p | *IGF1R* | hsa-miR-628-3p |
| *BCL2* | miR29a-3p  miR29b-3p | *IMPDH1* | miR29a-3p  miR29b-3p |
| *CCDC6* | hsa-miR-628-3p | *ITCH* | hsa-miR-628-3p |
| *CD274* | miR513a-5p | *ITGA11* | miR29a-3p |
| *CD276* | miR29a-3p | *KREMEN2* | miR29a-3p |
| *CDK6* | miR29a-3p  miR29b-3p | *LIN54* | hsa-miR-628-3p |
| *COL1A1* | miR29b-3p | *LPL* | miR29a-3p |
| *COL3A1* | miR29b-3p | *MCL1* | miR29a-3p  miR29b-3p |
| *COL4A1* | miR29a-3p  miR29b-3p | *MMP2* | miR29b-3p |
| *COL4A2* | miR29a-3p  miR29b-3p | *MMP24* | miR29b-3p |
| *COL5A3* | miR29a-3p  miR29b-3p | *MYCN* | miR29a-3p  miR29b-3p |
| *CPEB3* | miR29a-3p | *NCOA3* | miR29b-3p |
| *CPEB4* | miR29a-3p | *NEGR1* | hsa-miR-628-3p |
| *CRK* | hsa-miR-628-3p | *NID1* | miR29b-3p |
| *CTNNBIP1* | miR29b-3p | *NUFIP2* | hsa-miR-628-3p |
| *DCLK1* | hsa-miR-628-3p | *PIK3R1* | miR29a-3p |
| *DKK1* | miR29a-3p | *PLXNA1* | hsa-miR-628-3p |
| *DNAJB11* | miR29b-3p | *PPM1D* | miR29a-3p |
| *DNMT1* | miR29b-3p | *PTEN* | miR29a-3p  miR29b-3p |
| *DNMT3A* | miR29a-3p  miR29b-3p | *RAN* | miR29a-3p |
| *DNMT3B* | miR29a-3p  miR29b-3p | *RGS5* | hsa-miR-628-3p |
| *ESR1* | miR29b-3p | *S100B* | miR29a-3p  miR29b-3p |
| *FGA* | miR29a-3p  miR29b-3p | *SFPQ* | miR29b-3p |
| *FGB* | miR29a-3p  miR29b-3p | *SFRP2* | miR29a-3p |
| *FGG* | miR29a-3p  miR29b-3p | *SH3PXD2A* | hsa-miR-628-3p |
| *FOXO4* | hsa-miR-628-3p | *SP1* | miR29b-3p |
| *FRMPD4* | hsa-miR-628-3p | *TCL1A* | miR29b-3p |
| *GAPVD1* | hsa-miR-628-3p | *TNRC6B* | hsa-miR-628-3p |
| *GAS7* | hsa-miR-628-3p | *VEGFA* | miR29b-3p |
