# Supplementary material for: Burkitt lymphoma beyond MYC translocation: N-MYC and DNA methyltransferases dysregulation
Source: BMC Cancer. 2015 Oct 9;15:668. doi: 10.1186/s12885-015-1661-7 (PMC4600215; doi:10.1186/s12885-015-1661-7)
Supplement: Additional file 3: Table S3. — Gene set enrichment analysis for gene ontology categories of 64 genes predicted as targets of microRNAs differentially expressed in MYC translocation-positive and -negative BLs. (DOC 67 kb) [file 12885_2015_1661_MOESM3_ESM.doc]

**Supplementary Table 3.** **Gene set enrichment analysis for gene ontology categories of 64 genes predicted as targets of microRNAs differentially expressed in *MYC* translocation-positive and -negative BLs**

| **Gene Set Name** | **# Genes in Gene Set (K)** | **# Genes in Overlap (k)** | **k/K** | **FDR q-value** |
| --- | --- | --- | --- | --- |
| MULTICELLULAR_ORGANISMAL_DEVELOPMENT | 1049 | 14 | 0.0133 | 5.16E-08 |
| REGULATION_OF_GENE_EXPRESSION | 673 | 10 | 0.0149 | 4.82E-06 |
| NEGATIVE_REGULATION_OF_BIOLOGICAL_PROCESS | 677 | 10 | 0.0148 | 4.82E-06 |
| BIOPOLYMER_METABOLIC_PROCESS | 1684 | 13 | 0.0077 | 3.92E-05 |
| NEGATIVE_REGULATION_OF_DEVELOPMENTAL_PROCESS | 197 | 6 | 0.0305 | 3.92E-05 |
| DNA_MODIFICATION | 11 | 3 | 0.2727 | 4.14E-05 |
| CELL_PROLIFERATION_GO_0008283 | 513 | 8 | 0.0156 | 4.14E-05 |
| SYSTEM_DEVELOPMENT | 861 | 9 | 0.0105 | 1.61E-04 |
| REGULATION_OF_DEVELOPMENTAL_PROCESS | 440 | 7 | 0.0159 | 1.61E-04 |
| NEGATIVE_REGULATION_OF_CELLULAR_PROCESS | 646 | 8 | 0.0124 | 1.62E-04 |
| NUCLEOBASENUCLEOSIDENUCLEOTIDE_AND_NUCLEIC_ACID_METABOLIC_PROCESS | 1244 | 10 | 0.008 | 3.37E-04 |
| ONE_CARBON_COMPOUND_METABOLIC_PROCESS | 26 | 3 | 0.1154 | 3.70E-04 |
| ANATOMICAL_STRUCTURE_DEVELOPMENT | 1013 | 9 | 0.0089 | 4.06E-04 |
| MUSCLE_DEVELOPMENT | 93 | 4 | 0.043 | 4.14E-04 |
| REGULATION_OF_GENE_EXPRESSION_EPIGENETIC | 30 | 3 | 0.1 | 4.61E-04 |
| ORGAN_DEVELOPMENT | 571 | 7 | 0.0123 | 4.96E-04 |
| CELL_DEVELOPMENT | 577 | 7 | 0.0121 | 5.00E-04 |
| CELL_MATRIX_ADHESION | 38 | 3 | 0.0789 | 7.92E-04 |
| CELL_SUBSTRATE_ADHESION | 39 | 3 | 0.0769 | 8.12E-04 |
| REGULATION_OF_TRANSCRIPTIONDNA_DEPENDENT | 461 | 6 | 0.013 | 1.29E-03 |
| REGULATION_OF_RNA_METABOLIC_PROCESS | 471 | 6 | 0.0127 | 1.38E-03 |
| SIGNAL_TRANSDUCTION | 1634 | 10 | 0.0061 | 1.76E-03 |
| REGULATION_OF_CELL_PROLIFERATION | 308 | 5 | 0.0162 | 1.90E-03 |
| REGULATION_OF_CELLULAR_METABOLIC_PROCESS | 787 | 7 | 0.0089 | 2.48E-03 |
| IMMUNE_SYSTEM_PROCESS | 332 | 5 | 0.0151 | 2.48E-03 |
| REGULATION_OF_METABOLIC_PROCESS | 799 | 7 | 0.0088 | 2.58E-03 |
| REGULATION_OF_TRANSCRIPTION | 566 | 6 | 0.0106 | 2.92E-03 |
| CELLULAR_PROTEIN_METABOLIC_PROCESS | 1117 | 8 | 0.0072 | 2.92E-03 |
| CELLULAR_MACROMOLECULE_METABOLIC_PROCESS | 1131 | 8 | 0.0071 | 3.07E-03 |
| RNA_METABOLIC_PROCESS | 841 | 7 | 0.0083 | 3.07E-03 |
| PROTEOLYSIS | 191 | 4 | 0.0209 | 3.13E-03 |
| PHOSPHOLIPID_METABOLIC_PROCESS | 74 | 3 | 0.0405 | 3.31E-03 |
| REGULATION_OF_NUCLEOBASENUCLEOSIDENUCLEOTIDE_AND_NUCLEIC_ACID_METABOLIC_PROCESS | 618 | 6 | 0.0097 | 3.90E-03 |
| TRANSCRIPTION_DNA_DEPENDENT | 636 | 6 | 0.0094 | 4.37E-03 |
| RNA_BIOSYNTHETIC_PROCESS | 638 | 6 | 0.0094 | 4.37E-03 |
| PROTEIN_METABOLIC_PROCESS | 1231 | 8 | 0.0065 | 4.42E-03 |
| BIOPOLYMER_MODIFICATION | 650 | 6 | 0.0092 | 4.57E-03 |
| INSULIN_RECEPTOR_SIGNALING_PATHWAY | 19 | 2 | 0.1053 | 6.13E-03 |
| MEMBRANE_LIPID_METABOLIC_PROCESS | 101 | 3 | 0.0297 | 6.81E-03 |
| DNA_METABOLIC_PROCESS | 257 | 4 | 0.0156 | 7.51E-03 |
| TRANSCRIPTION | 753 | 6 | 0.008 | 9.01E-03 |
| ANTI_APOPTOSIS | 118 | 3 | 0.0254 | 9.97E-03 |
| REGULATION_OF_TRANSCRIPTION_FROM_RNA_POLYMERASE_II_PROMOTER | 289 | 4 | 0.0138 | 1.09E-02 |
| PHOSPHOINOSITIDE_METABOLIC_PROCESS | 31 | 2 | 0.0645 | 1.43E-02 |
| CELL_CYCLE_GO_0007049 | 315 | 4 | 0.0127 | 1.43E-02 |
| POSITIVE_REGULATION_OF_CELL_PROLIFERATION | 149 | 3 | 0.0201 | 1.75E-02 |
| NEGATIVE_REGULATION_OF_APOPTOSIS | 150 | 3 | 0.02 | 1.75E-02 |
| NEGATIVE_REGULATION_OF_PROGRAMMED_CELL_DEATH | 151 | 3 | 0.0199 | 1.75E-02 |
| REGULATION_OF_APOPTOSIS | 341 | 4 | 0.0117 | 1.75E-02 |
| REGULATION_OF_PROGRAMMED_CELL_DEATH | 342 | 4 | 0.0117 | 1.75E-02 |
